# Influence of N-terminal His-tags on the production of recombinant proteins in the cytoplasm of *Bacillus subtilis*

**DOI:** 10.1016/j.btre.2022.e00754

**Published:** 2022-07-19

**Authors:** Ngan Thi Phuong Le, Trang Thi Phuong Phan, Hanh Thi Thu Phan, Tuom Thi Tinh Truong, Wolfgang Schumann, Hoang Duc Nguyen

**Affiliations:** a Center for Bioscience and Biotechnology, University of Science, Ho Chi Minh City, Vietnam; b Vietnam National University, Ho Chi Minh City, Vietnam; c Laboratory of Molecular Biotechnology, University of Science, Ho Chi Minh City, Vietnam; d Cancer research laboratory, University of Science, Ho Chi Minh City, Vietnam

**Keywords:** His-tag, Fusion tag, P*grac*212 promoter, *Bacillus subtilis*, Low expression gene

## Abstract

The influence of fusion tags to produce recombinant proteins in the cytoplasm of *Bacillus subtilis* is not well-studied as in *E. coli*. This study aimed to investigate the influence of His-tags with different codons on the protein production levels of the high expression gene (*gfp+*) and low expression gene (*egfp*) in the cytoplasm of *B. subtilis* cells. We used three different N-terminal His-tags, M-6xHis, MRGS-8xHis and MEA-8xHis, to investigate their effects on the production levels of GFP variants under the control of the P*grac*212 in *B. subtilis*. The fusions of His-tags with GFP+ caused a reduction compared to the construct without His-tag. When three His-tags fused with *egfp*, the EGFP production levels were significantly increased up to 3.5-, 12-, and 15-fold. This study suggested that His-tag at the N-terminus could enhance the protein production for the low expression gene and reduce that of the high expression gene in *B. subtilis*.

## Highlights

• N-terminus His-tags influence on gene expression in the cytoplasm of *B. subtilis*.
• His-tags decrease the protein production of the highly expressed genes.
• His-tags could enhance the fusion protein production of the low expression gene.

## 1 Introduction

*B. subtilis* offers many advantages for use in the production of recombinant proteins. Unlike the Gram-negative *E. coli, B. subtilis* is generally recognized as safe (GRAS) because it is non-pathogenic and nontoxic [1,2]. In addition, *B. subtilis* is well known for its high capacity to secrete proteins [3]. Many modifications of the host strain improved the secretion of recombinant protein production [4]. The expression systems also developed in *B. subtilis* [5]. Most studies on the intracellular production of recombinant proteins have focused on *B. subtilis* [6] using promoter elements optimized for strong expression [7]. Vectors based on P*grac*100 were developed that showed high protein production levels in *B. subtilis* and decreased basal expression in *E. coli* [8]. Modifications of IPTG-inducible promoters have resulted in inducer-free expression plasmids [9], while some reporter genes have been successfully expressed in the cytoplasm of *B. subtilis* like BgaB [7], [8], [9], GFP [7,9], and GUS [10]. An optimal combination of strong promoters, transcription terminators, and various translation/secretion signals can achieve a high protein expression level [11]. In addition, the fusion tag also significantly affects the production levels of intracellular proteins.

Fusion tags are short peptides or proteins attached to the N- or C-terminus of the target protein to enhance production, increase solubility and facilitate its purification [12]. Fusion tags include short sequences such as polyhistidine (poly-His), polyarginine (poly-Arg), FLAG, c-myc, or Strep-tag, and proteins such as maltose-binding protein (MBP), glutathione S-transferase (GST), N-utilization substance A (NusA), thioredoxin (Trx), small ubiquitin-related modifier (SUMO) [13,14]. The larger tags are often used to increase the solubility of target proteins [15]. It is known that the fusion tag has an effect on protein production in *E. coli*. The position, sequence, and length of the fusion tag can affect protein production on several levels, including production levels, solubility, binding to the immobilized metal affinity chromatography (IMAC) ligand, tertiary structure, propensity to form crystals, and activity [16,17]. The nucleotide sequence around the translation initiation region (TIR) also substantially impacts translation efficiency in prokaryotes [13]. However, a system that optimizes both production and purification of target proteins in *B. subtilis* has not been developed yet.

A His-tag containing 2–10 histidine residues (commonly 6xHis) is the fusion tag most frequently used because of its small size (about 0.84 kDa for 6xHis). In *E. coli*, a His-tagged recombinant protein can be purified by IMAC under denaturing conditions and refolded if it is insoluble [18]. His-tagging offers the advantage of a simple, one-step purification using inexpensive materials. However, some studies have shown that His-tags are related to insolubility and can affect the structure and function of the target protein [19]. An earlier investigation reported that an N-terminal polyhistidine tag can influence the thermal stability of the recombinant protein [20]. Our previous work on His-tagged BgaB and GFP proteins confirmed that production levels of these recombinant proteins were significantly reduced when they were fused with N-terminal histidine residues [8,21,22]. Therefore, selecting a His-tag with an appropriate sequence for protein production is still a major challenge.

This study aimed to provide primary data to understand the influence of His-tags on the protein production levels of the high and low expression genes in *B. subtilis*. We evaluated the effectiveness of existing and predicted His-tag DNA sequences on the cytoplasmic production of recombinant proteins under the control of the strong promoter, P*grac*212 [23,24], in B. subtilis. Target proteins included high-expression gene sequences optimized for the expression in *B. subtilis*, GFP+ [25], and a low-expression gene sequence coding for the EGFP with codons optimized for mammalian cells [26]. We compared production levels of the target proteins fused with different His-tag sequences and evaluated their purity after single-step purification on a spin column.

## 2 Materials and methods

### 2.1 Bacterial strains, plasmids, oligonucleotides, and growth conditions

The plasmids and oligonucleotides used in this study are shown in Table 1 and Table 2. The *E. coli* strain OmniMAX from Invitrogen was used for the cloning experiments, and *B. subtilis* 1012 [27] obtained from MoBiTec was used for the production of proteins. Cells were grown in Luria broth (LB) at 37 °C with shaking. The antibiotics, ampicillin at 0.1 g *l*^−1^ for *E. coli* and chloramphenicol at 0.01 g *l*^−1^ for *B. subtilis*, were added to the culture media.

**Table 1:** The plasmids used in this study.

**Plasmid**	**Description**	**Source/reference**
pHT100	P*grac*100-*bga*B	[7]
pHT100-gfp+	P*grac*100-*gfp*+	[8]
pHT1025	P*grac*212-*egfp*	This study
pHT1026	P*grac*212-MEA-8xHis-*egfp*	This study
pHT1066	P*grac*212-*gfp*+	From lab collection
pHT1070	Containing *egfp* gene originated from pEGFP-N1 of Clontech; using as a template to aplify *egfp* gene	This study
pHT1169	P*grac*100-MEA-8xHis-*gfp*	[8]
pHT1178	P*grac*100-MEA-8xHis-*bga*B	[8]
pHT1222	P*grac*212-MCS-*gfp*+DBamHI (high copy number); Amplifying *gfp+* gene	This study
pHT1259	P*grac*212-His-thrombin-MCS-Strep (high copy); used to construct pHT1262, pHT2472	This study
pHT1262	P*grac*212-M-6xHis-*egfp*	This study
pHT1266	P*grac*212-MRGS-8xHis-MCS-Strep; used to construct pHT2466	This study
pHT1611	P*grac*212-MRGS-8xHis-*bga*B	This study
pHT212	P*grac*212-*bga*B	[23]
pHT2466	P*grac*212-MRGS-8xHis-*egfp*	This study
pHT2472	P*grac*212-M-6xHis-*gfp*+	This study
pHT2473	P*grac*212-MEA-8xHis-*gfp*+	This study
pHT2474	P*grac*212-MRGS-8xHis-*gfp*+	This study
pHT259	P*grac*212-MEA-8xHis-MCS, originated from pHT212; used to construct pHT1026, pHT2473	This study
pHT261	P*grac*212-MCS-Strep-Tag, originated from pHT212; used to construct pHT1025	This study

**Table 2:** The oligonucleotides used in this study.

**Oligonucleotide**	**Sequence, 5′→ 3′**	**Used for**
ON1277	AAAGGAGGAAGGATCCATGGCTAGCAAAGGAGAAGAACT	Amplifying *gfp*+ gene; constructing pHT2472, pHT2473, pHT2474
ON742	TAGGCGGGCTGCCCCGGGTTATTTGTAGAGCTCATCCATGCCATGTG	Amplifying *gfp*+gene, constructing pHT2472, pHT2473, pHT2474; colony PCR pHT2472, pHT2473, pHT2474
ON1359	ACGTACGATCTTTCAGCCGACTC	Colony PCR pHT2472, pHT2473, pHT2474, pHT2466
ON1375	GTTTCAACCATTTGTTCCAGGTAAG	Sequencing pHT2472, pHT2473, pHT2474, pHT2466
ON549	GTACTTCCAGGGATCCATGGTGAGCAAGGGCGAGGAGCTG	Amplifying *egfp* gene; constructing pHT1025, pHT1026, pHT2466; colony PCR pHT1025, pHT1026
ON632	TAGGCGGGCTGCCCCGGGGACG	Amplifying *egfp* gene; constructing pHT1025, pHT1026, pHT2466; colony PCR pHT2466
ON227	GGTGCCACGCGGATCTGTGAGCAAGGGCGAGGAGCTG	Amplifying *egfp* gene to construct pHT1262
ON228	CGACGTCGACTCTAGAGATCCCGGCGGCGGTCACG	Amplifying *egfp* gene; constructing pHT1262; colony PCR pHT1262
ON653	ACCGGAATTAGCTTGGTACCAGCTATTG	Sequencing pHT1025 and pHT1026; colony PCR pHT1262
ON314	TGTTTCAACCATTTGTTCCAGGT	Sequencing pHT1025 and pHT1026; colony PCR pHT1025, pHT1026, pHT1262

### 2.2 Construction of recombinant plasmids

To investigate the influence of the different His-tags on protein production, target proteins were selected, and expression plasmids were designed with an His-tag. For pHT1611, the His-tag sequence was generated by hybridization of the complementary ON1989 ON1990 oligonucleotides. The hybridization product was cut with *Bam*HI and cloned into plasmid pHT212 at the *Bam*HI site. In constructing pHT1025, pHT1026 and pHT2466, the *egfp* gene was first amplified from the plasmid pHT1070 by polymerase chain reaction (PCR) using the primers ON549 and ON632. The PCR products were cut with *Bam*HI/*Sma*I and cloned into the plasmids, pHT261, pHT259, and pHT1266 at the *Bam*HI and *Sma*I sites. To construct pHT1262, the e*gfp* gene was amplified using the primer pairs ON227 and ON228, with pHT1070 as a template, and the PCR products were introduced into pHT1259 at the *Bam*HI and *Xba*I sites. For pHT2472, pHT2473, and pHT2474, the *gfp+* gene was amplified using the primer pairs ON1277 and ON742, with pHT1222 as a template. The PCR products were cut with *Bam*HI/*Sma*I and cloned into pHT1259, pHT259, and pHT1266 at the *Bam*HI and *Sma*I sites.

### 2.3 The production of recombinant proteins

The plasmids were introduced into *B. subtilis* 1012 by natural transformation [28]. Recombinant *B. subtilis* strains were streaked onto LB agar containing chloramphenicol and incubated overnight at 37 °C. A single colony was inoculated into 10 ml LB medium with chloramphenicol and shaken overnight at 37 °C. Cultures of each strain were replicated using two separate colonies. An appropriate volume of an overnight culture of each clone was transferred to 30 ml LB containing chloramphenicol to give an optical density at 600 nm (OD_600_) of 0.1 and incubated under shaking at 37 °C. When the OD_600_ of the culture reached 0.8–1, the cells were divided into three sub-cultures, and two were induced by the addition of IPTG to a final concentration of 0.1 and 1 mM, respectively. Cells were collected at 0 h just before induction and at 2 and 4 h after induction by centrifugation at 6000 *g* for 10 min at 4 °C. The number of *B. subtilis* cells sampled was equivalent to those present in 1 ml of culture with an OD_600_ of 2.4. Samples were prepared for target protein measurement by fluorescence and SDS-PAGE analysis.

### 2.4 Measurement of green fluorescent protein production in B. subtilis

Cells were lysed by incubation in 500 μl PBS containing 0.2 g *l*^−1^ lysozyme at 37 °C for 30 min. Lysates were centrifuged at 12,000 *g* for 5 min and protein fluorescence was measured in a black microtiter plate (Nunc) using a microplate reader with an excitation wavelength of 465 (+/−8) nm and an emission wavelength of 510 (+/−8) nm for EGFP and excitation at 470 (+/−8) nm and emission at 515 (+/−8) nm for GFP+. The experiments were carried out with two different colonies, and standard errors were calculated.

### 2.5 SDS-PAGE and western immunoblotting

For SDS-PAGE analysis, cells were grown as described above for 4 h in the presence of different concentrations of IPTG (0, 0.1 or 1 mM), and samples taken at an OD_600_ of 2.4 were collected by centrifugation at 6000 *g* for 10 min at 4 °C. The pellets were resuspended in 100 µl of the lysis PBS buffer, containing 1.25 g *l*^−1^ lysozyme, and incubated at 37 °C for 5 min, after which 25 µl of 5X sample loading buffer was added to each lysate. After heating at 95 °C for 5 min, the samples were centrifuged at 12,000 *g* for 5 min and supernatants were loaded into an SDS-PAGE gel (12% polyacrylamide). The separated proteins were transferred from the gel to a nitrocellulose membrane, blocked with 5% skim milk in PBS-T (PBS with 0.1% Tween 20) for 1 h, then incubated with primary mouse anti-GFP serum at the dilution 1:10,000 at room temperature for 1 h. After washing with PBS-T, the nitrocellulose membrane was incubated with HRP-conjugated anti-mouse IgG secondary antibody for 1 hour at room temperature and washed with PBS-T. For detecting the EGFP protein, the nitrocellulose membrane was incubated with TMB (tetramethylbenzidine), according to the manufacturer's procedure and the protein bands were imaged with a scanner. The density of the target protein was determined using AlphaEase FC 4.0 software and the relative target protein production was calculated by densitometry.

### 2.6 Purification of recombinant proteins using Ni-NTA spin columns

*B. subtilis* 1012 carrying different plasmids was grown in LB to mid-log phase, and production of the recombinant proteins was induced by the addition of 1 mM IPTG. The cells were collected by centrifugation, resuspended in lysis buffer with 0.02 g l-1 lysozyme, and disrupted by sonication. The protocol with recommended buffers for Ni-NTA spin column (Qiagen) was followed, with washing buffer containing 20 mM imidazole and elution buffer containing 500 mM imidazole.

## 3 Results

### 3.1 Does an N-terminal His-tag reduce the production of BgaB and GFP+?

**Fig. 1:**
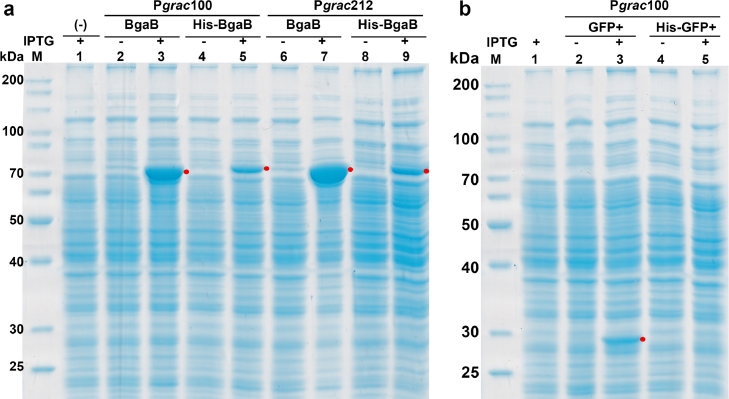
Production of BgaB and GFP+ proteins with and without N-terminal His-tag in *B. subtilis*. **(a)** pHT100 (P*grac*100-*bga*B); pHT1178 (P*grac*100-His-*bga*B), pHT212 (P*grac*212- *bga*B) and pHT1611 (P*grac*212-His-*bga*B); **(b)** pHT100-*gfp* (P*grac*100-*gfp*); pHT1169 (P*grac*100-MEA-8xHis-*gfp*). Samples were harvested at 4 h after induction with IPTG at 0 mM (-) and 1 mM (+).

In the previous study, a His-tag at the N-terminus (P*grac*100-*his*-*bga*B) drastically reduced the production of BgaB as compared to P*grac*100-*bga*B without a His-tag [8]. We used the P*grac*212 promoter, which is stronger than P*grac*100, to evaluate the level of the fusion protein His-BgaB, and found that the protein production from P*grac*212-*his*-*bgaB* (**pHT1611**) was equivalent to that of the P*grac*100-*his*-*bgaB* construct (**pHT1178**) (Fig. 1a). In the absence of a His-tag, the stronger promoter P*grac*212 in **pHT212** produced a higher BgaB production level than **pHT100**. However, fusion with an N-terminal His-tag significantly reduced BgaB production by up to 60%, confirming the negative effect of His-tag fusion at the N-terminus on production of the BgaB protein.

Like BgaB, GFP+ can be highly overexpressed in *B. subtilis*. In the previous study, the N-terminal His-tag also decreased the production of the GFP+ protein as compared to the C-terminal His-tag [8]. The production in B. subtilis of the GFP+ protein with N-terminal His-tag under the control of the Pgrac100 promoter (**pHT1169**) was dramatically reduced as compared to **pHT100-*gfp*** without a His-tag, and the target protein band was hard to be seen on an SDS-PAGE gel (Fig. 1**b**). The decrease in the GFP+ level when fused with a His-tag at the N-terminus was similar to that of the BgaB protein.

### 3.2 Evaluation of the effect of different His-tag sequences on the production of the GFP+ protein

**Fig. 2:**
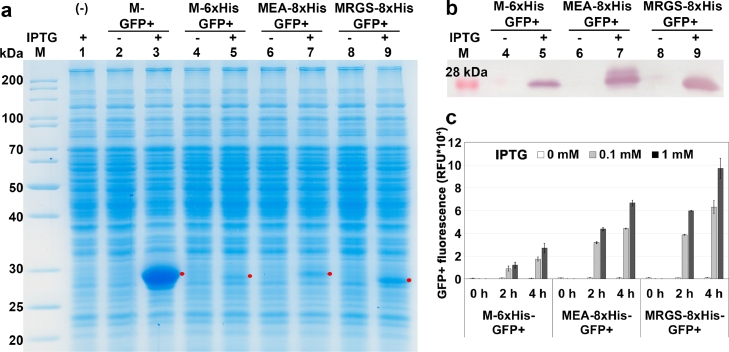
Production of GFP+ protein fused with different His-tags in *B. subtilis*: pHT2472 (P*grac*212-M-6xHis-*gfp*+); pHT2473 (P*grac*212-MEA-8xHis-*gfp*+) and pHT2474 (P*grac*212-MRGS-8xHis-*gfp*+). Samples were harvested at 4 h after induction with IPTG at 0 mM (-) and 1 mM (+): **(a)** SDS-PAGE; **(b)** Western blot. **(c)** GFP+ fluorescence intensity. Samples were harvested at 0 h (when the OD_600_ reached 0.8–1), 2 h and 4 h after induction with IPTG at 0 mM, 0.1 mM and 1 mM.

The translation efficiency of prokaryotes is influenced by the nucleotide sequence around the TIR [13]. In this study, we tested three different His-tag sequences, M-6xHis, MRGS-8xHis and MEA-8xHis (Table 3) and compared their effects on GFP+ production under the control of P*grac*212. We generated three plasmids, **pHT2472 (**P*grac*212-M-6xHis-*gfp*+), **pHT2473** (P*grac*212-MEA-8xHis-*gfp*+) and **pHT2474** (P*grac*212-MRGS-8xHis-*gfp*+) and transformed them into competent *B. subtilis* cells. Quantification of His-GFP+ production was carried out by SDS-PAGE (Fig. 2a) and western blot (Fig. 2b). These results confirmed that all three His-tag sequences significantly reduced GFP+ production by about 73% as compared to the control without a His-tag. Analysis of the target protein band density compared to total cellular proteins revealed that MRGS-8xHis-*gfp*+ (**pHT2474**) had the highest production level (about 9.4% of the total cellular proteins) and was 2.5 times as high as M-6xHis-*gfp*+ (**pHT2472**).

**Table 3:** Properties of His-tagged peptides.

**His-tag**	**DNA sequence**	**Peptide sequence**	Length (aa)*	Molecularweight (Da)*
M-6xHis	atgcaccatcatcatcatcattcttctggtctggtgccacgcggatcc	MHHHHHHSSGLVPRGS	16	1811.85
MRGS-8xHis	atgaggggaagccatcaccatcaccatcaccatcacggatcc	MRGSHHHHHHHHGS	14	1689.73
MEA-8xHis	atggaagctcatcaccatcaccatcaccatcacggatcc	MEAHHHHHHHHGS	13	1589.65

⁎ Determined by using the PepDraw tool (www.pepdraw.com).

In Fig. 2c, GFP+ fluorescence increased with increasing IPTG concentrations from 0 mM to 1 mM and increasing induction time from 0 h to 4 h. Comparing the green fluorescence intensity level of GFP+ proteins fused with different His-tags at 4 h and 1 mM IPTG, **pHT2474** (MRGS-8xHis) showed the highest level and is 3.5 times as high as the amount of **pHT2472** (M-6xHis). Among three His-tagged sequences (Table 3) fused to the GFP+ N*-*terminus, the MRGS-8xHis sequence yielded the highest GFP+ production level. Comparing fluorescence of GFP+ proteins fused with different His-tags at 4 h and 1 mM IPTG, **pHT2472** (M-6xHis), pHT2473 (MEA-8xHis), pHT2474 (MRGS-8xHis) showed the green fluorescence intensity lower than pHT1066 (without His-tag) 35, 14 and 10 times, respectively.

### 3.3 Enhancing the production of EGFP by fusion with different His-tags

**Fig. 3:**
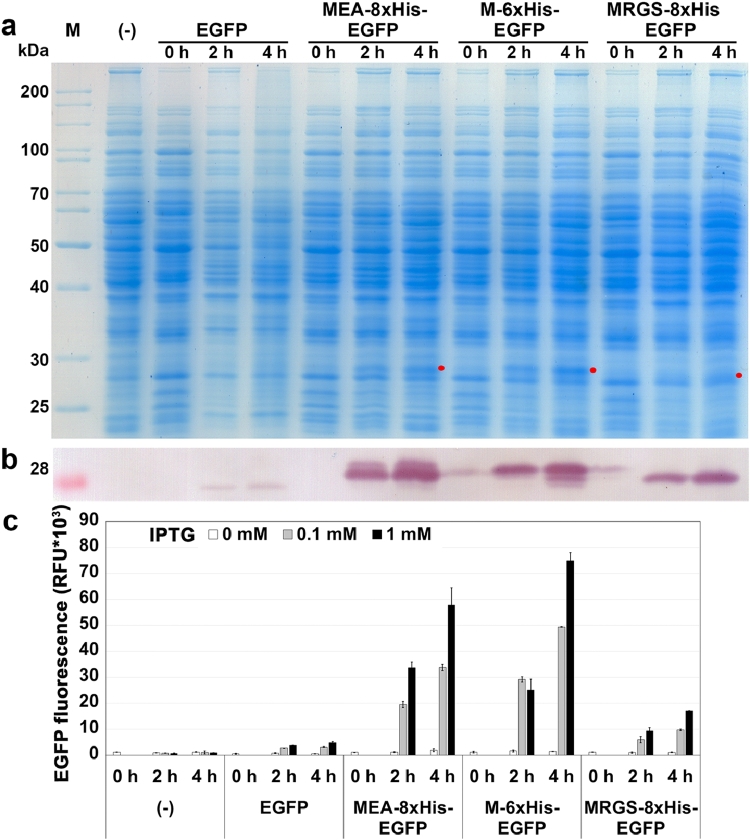
Production of EGFP protein fused with different His-tags in *B. subtilis*: pHT1026 (P*grac*212-MEA-8xHis-*egfp*); pHT1062 (P*grac*212-His-*egfp*) and pHT2466 (P*grac*212-MRGS-8xHis-*egfp*). Samples were harvested at 0 h (when the OD_600_ reached 0.8–1) and 2 h and 4 h after induction with IPTG at 0 mM (-) and 1 mM (+). **(a)** SDS-PAGE, **(b)** Western blot, **(c)** EGFP fluorescence intensity. Samples were harvested at 0 h (when the OD_600_ reached 0.8–1), 2 h and 4 h after induction with IPTG at 0 mM, 0.1 mM and 1 mM.

*egfp* is a *gfp* variant that has been codon-optimized for high expression level in mammalian cells [26]. In this study, we designed plasmids containing the *egfp* gene fused to the three His-tag sequences as shown above (Table 3) and evaluated their effect on EGFP protein production. We generated four plasmids: **pHT1025** (P*grac*212-*egfp*), **pHT1262 (**P*grac*212-M-6xHis-*egfp*), **pHT1026** (P*grac*212-MEA-8xHis-*egfp*) and **pHT2466** (P*grac*212-MRGS-8xHis-*egfp*), which were then transformed into *B. subtilis* and expressed. The His-EGFP protein was detected by SDS-PAGE (Fig. 3a) and Western blot (Fig. 3b). The production of the EGFP protein without His-tag after incubation with 1 mM IPTG was low, and the EGFP protein band was not visible on a stained SDS-PAGE gel, but it was seen by Western immunoblotting. All three His-tags, M-6xHis, MEA-8xHis, and MRGS-8xHis, significantly increased production of EGFP as compared to the control without His-tag (**pHT1025**).

In Fig. 3c, EGFP fluorescence increased with His-tag fusion and with increasing IPTG concentration from 0 mM to 1 mM and induction time from 0 h to 4 h. Comparing the fluorescence of EGFP proteins fused with different His-tags at 4 h and 1 mM IPTG, **pHT1262** (M-6xHis) showed the highest green fluorescence intensity and was 15 times higher than **pHT1025** without His-tag.

Each of the three His-tags increased the EGFP production to a level detectable on a stained SDS-PAGE gel. However, the production and fluorescence levels of EGFP differed with the different His-tags. The M-6xHis sequence resulted in the highest EGFP production.

### 3.4 Effect of adding histidine to the culture medium on the production of N-terminal His-tagged proteins

**Fig. 4:**
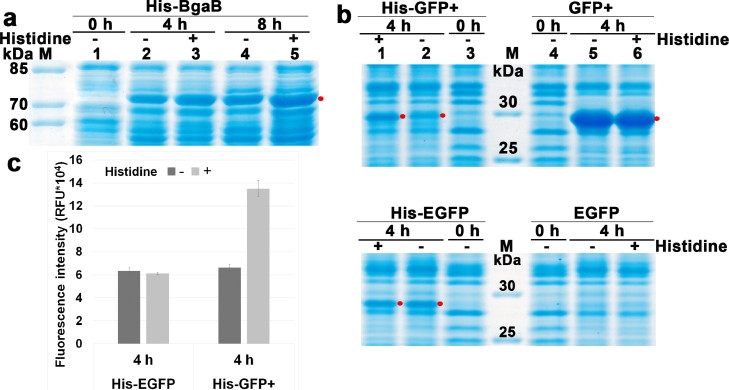
Production of BgaB, GFP+ and EGFP fused with N-terminal His-tag with 50 mM histidine added to the culture medium (+) or no histidine (-). **(a)** Stained SDS-PAGE gel showing production of BgaB, GFP+ and EGFP proteins (*red dot*) fused with His-tag: pHT1611 (P*grac*212-His-*bga*B); pHT2473 (P*grac*212-His-*gfp+*); pHT1026 (P*grac*212-His-*egfp*). **(b)** Fluorescence intensity of His-EGFP (pHT1026) and His-GFP+ (pHT2473). Samples were harvested at 0 h (OD_600_ of 0.8 to 1.0), 4 h and 8 h after induction with 1 mM IPTG.

The lower production level of the BgaB and GFP+ proteins with an N-terminal poly-His-tag could be due to a deficiency in His-at the initial phase of elongation, which would reduce the speed and efficiency of translation through diminished loading of tRNA^His^, limiting the transport of His-to the ribosome. The decreased supply of histidine could result in lower production of the target protein. To see if this reduction in production was due to histidine deficiency during poly-His-synthesis, we added histidine to the culture medium (Fig. 4a). The results in Fig. 4a showed that when histidine (+) was added to the culture medium and transcription induced by 1 mM IPTG, the production of His-BgaB was higher than in the absence of histidine (-). Analysis of the target protein bands using AlphaEase 4.0 software, showed that adding histidine to the culture medium increased the production level of the target protein by 32% after 4 h of IPTG induction as compared to the no-His-control. Thus, the decrease in BgaB protein with His-tag at the N-terminus was partly due to the lack of histidine during the initial phase of elongation. The experiment was repeated with the His-GFP+ and His-EGFP compared to the controls without His-tag (Fig. 4b). The fluorescence intensity of His-GFP+ and His-EGFP after 4 h of 1 mM IPTG induction was measured, and the results are shown in Fig. 4c. The *gfp*+ and *egfp* without His-tag showed no difference in expression level on SDS-PAGE with (+) or without (-) histidine addition to the culture medium. Similar to His-BgaB, His-GFP+ increased the expression level and fluorescence intensity when histidine was added. In contrast, for the low-expression gene in *B. subtilis*, His-tag fusion at the N-terminus increased the expression level of the EGFP protein, and the addition of histidine to the culture medium did not affect His-EGFP expression levels.

### 3.5 Purification of recombinant proteins

**Fig. 5:**
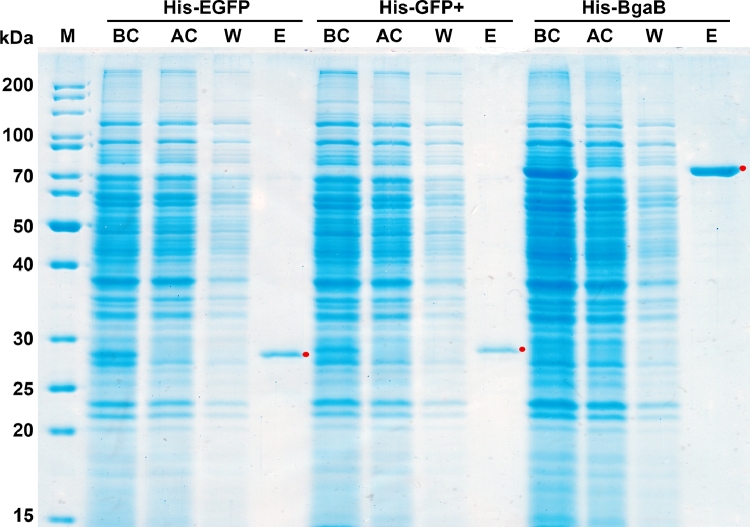
Affinity purification of proteins with N-terminal His-tag expressed in *B. subtilis. B. subtilis* 1012 carrying pHT1026 (His-EGFP), pHT2473 (His-GFP+), and pHT1611 (His-BgaB) were grown in LB medium. Samples were harvested at 4 h after induction with 1 mM IPTG, the cells were lysed and extracts were run on nickel-NTA columns. **BC** (before column), **AC** (after column), **W** (wash), and **E** (eluate). The purified protein bands are indicated by the red dot.

To determine whether His-tagged recombinant proteins produced in *B. subtilis* can be purified via one-step purification, we used Ni-NTA spin columns and chose *B. subtilis* strains containing plasmids **pHT1026** (His-EGFP); **pHT2473** (His-GFP+), and **pHT1611** (His-BgaB). These strains were cultured for 4 h with 1 mM IPTG and cells were harvested by centrifugation at 6000 *g* for 10 min at 4 °C. The before-column, after-column, washes and eluted fractions were analyzed by SDS-PAGE (Fig. 5) and densitometry was done on the target protein bands in the eluted fraction using the AlphaEase 4.0 software. The purity of His-EGFP, His-GFP+, and His-BgaB recombinant proteins was greater than 88%. The result proved that proteins fused with His-tag expressed in *B. subtilis* could be easily purified via a single step.

## 4 Discussion

His-tag fusions with the target proteins were used to facilitate purification, but they could interfere with the protein production levels. Fusion tags can be used at the N-terminus or the C-terminus of recombinant proteins. Our previous reports showed that GFP and BgaB proteins with C-terminal His-tag were at higher levels than that of proteins with N-terminal His-tag in *B. subtilis* [8,21,22]. Another paper indicated that His-tag position at C-terminus could increase the yield and activity of CotA protein [29]. These examples are a few pieces of evidence for the successfulness of producing recombinant proteins with high expression genes fused with His-tag in *B. subtilis*. Surprisingly, these His-tag fusions at the N-terminus reduced the protein production levels compared with the His-tag fusion at the C-terminus. We asked what happened if His-tag was fused at the N-terminus with the low expression gene in comparison with the high expression gene. This study showed that His-tags fused at the N-terminus reduced the protein production levels of high expression genes (*bgaB* and *gfp+*) while enhancing the protein production levels of the low expression gene (*egfp*) in *B. subtilis*.

The results in this study confirmed a previous report that the DNA sequence at the N terminus affected transcription and initiation of translation [8]. The TIR sequence promotes the interaction with rRNA that initiates translation [30]. Prokaryotic translation efficiency is most affected by the folding free energy of the region between nucleotides -10 and +35. Therefore, to achieve high-production protein levels, the nucleotide sequence around the TIR [31] needs to be optimized. We designed His-tags attached to the N-terminus of target proteins and then evaluated the effect of DNA encoding the His-tags with existing and predicted sequences on the production of recombinant proteins. The *gfp*+ has codons optimized for expression in prokaryotic cells and can be used for high production in *B. subtilis*. Fusing three different sequences of multiple codons for repeated histidine residues at the N-terminus of the high-production GFP+ protein resulted in reduced GFP+ production.

Since the N-terminal coding sequences influences the efficiency of ribosome binding to the mRNA and ribosome extension at the initial stage of translation, it strongly affects gene expression at the level of translation [32]. At the initial phase of elongation, the synthesis of poly-histidine affects the efficiency of translation. Adding histidine to the culture medium increased production of BgaB and GFP+ with N-terminal His-tags. Therefore, the decrease in production of the high-expression genes is partly due to the lack of histidine during the initial phase of elongation. Increasing the number of histidine-coding codons affects the initiation of elongation, possibly due to unfavorable transport of the histidine amino acids for protein synthesis, causing decreased GFP+ production. The influence of the second amino acid on recombinant protein production was studied in *E. coli* [33]. It was shown that Met, His, and Glu-at the +2 position resulted in greatly reduced protein production of recombinant Igα. M-6xHis-*gfp*+ (**pHT2472**) has His-at the +2 position and showed the lowest production level among the three His-tag sequences. MRGS-8xHis (**pHT2474**) showed the highest GFP+ production level that was 3.5 times as high as **pHT2472** (M-6xHis).

For the *egfp* gene with codons optimized for mammalian cells, the production level of EGFP in *B. subtilis* was low. This study clearly indicated that His-tag could enhance the production of the recombinant protein for the low expression gene such as *egfp* in *B. subtilis*. In the control (pHT1025) carrying the *egfp* gene, the target protein band could not be detected by SDS-PAGE but was seen by Western immunoblotting. However, the EGFP fused with the His-tag at the N-terminus increased production of this protein significantly up to fifteen times higher than the control without His-tag. The most exciting experimental design for the study is using two *gfp* genes with different sequences, *gfp*+, optimized for bacteria with CAI 0.759 for *B. subtilis* and *egfp*, optimized for mammalian with CAI 0.637 for *B. subtilis*. The distinction in the gene optimization leads to higher GFP+ production levels in *B. subtilis* than EGFP. Based on the effect of His-tag in the N-terminus, we found that there is a difference between these two representatives. His-tags at the N-terminus reduced the protein synthesis of the highly expressed protein (GFP+) and increased the protein production of the low expression protein (EGFP) in *B. subtilis* but not in *E. coli* (data not shown).

The analysis results of codon adaptation index (CAI) *B. subtilis* by using the “CAI calulator” showed in Table 4. The CAI index ​​are about 0.024 apart between the whole sequences of *egfp* gene and *egfp* fusion with His-tags. However, the CAI index of the first 10 codons has a big difference of about 0.199 between these genes. It is likely that the *B. subtilis* CAI index increased with His-tag sequence, and the higher the CAI for the first 10 codons, the greater the EGFP synthesis in *B. subtilis*. The codon choice could affect protein synthesis in *B. subtilis* and increase the production of the low expression protein such as EGFP.

**Table 4:** Analysis of the codon adaptation index (CAI) for *B. subtilis* and secondary structure energy of mRNA.

**Plasmid**	**Characteristic**	CAI of the whole fusion gene*	CAI of the first 10 codons*	SS (RNAfold for the first 10 codons) (J/mol)*		**Expression level**
pHT1025	EGFP	0.637	0.638		- 11.5	Control of EGFP
pHT1026	MEA-8xHis-EGFP	0.641	0.771		- 0.7	12-fold increase
pHT1262	M-6xHis- EGFP	0.661	0.837		- 0.6	15-fold increase
pHT2466	MRGS-8xHis-EGFP	0.64	0.699		- 3.3	3.5-fold increase
pHT1066	GFP+	0.759	0.81		−1.5	Control of GFP+

⁎ Determined by using the CAI calculator and 10-codon RNAfold tool.

The translation process is influenced by the secondary structure of the mRNA [34], and some studies have shown that secondary structure in the 5′ untranslated region (5′ UTR) generally reduces translation initiation efficiency and overall protein production [35]. The more negative the secondary structure energy, the greater the formation of the secondary structure. This affects the ribosomes’ binding to and moving on the mRNA during translation. We analyzed the secondary structure (SS) energy at the first ten codons of mRNA in these sequences by using the **RNAfold** tool, and the results are shown in Table 4. The secondary structural energies were proportional to the production levels of EGFP proteins fused with different His-tag sequences. These results showed that His-tag affects the expression of low expression genes in *B. subtilis* and tends to be optimal for expression in *B. subtilis*.

His-tagged recombinant proteins in *B. subtilis* can be purified using Ni-NTA spin columns with an efficiency >88%. However, the fusion of a His-tag had a significant effect on the production levels of BgaB, GFP+, and EGFP proteins. Different His-tag sequences have different effects on the production levels of fusion proteins. Based on our results, His-tag at the N-terminus could enhance the protein production for the low expression gene in *B. subtilis* and reduce protein production for high expression genes.

## CRediT authorship contribution statement

**Ngan Thi Phuong Le:** Investigation, Methodology, Data curation, Writing – original draft. **Trang Thi Phuong Phan:** Conceptualization, Writing – review & editing. **Hanh Thi Thu Phan:** Methodology, Data curation. **Tuom Thi Tinh Truong:** Methodology. **Wolfgang Schumann:** Writing – review & editing, Supervision. **Hoang Duc Nguyen:** Conceptualization, Writing – review & editing, Project administration.

## Declaration of Competing Interest

The authors declare that they have no known competing financial interests or personal relationships that could have appeared to influence the work reported in this paper.
